# Collective agency and the concept of ‘public’ in public involvement: A practice-oriented analysis

**DOI:** 10.1186/s12910-015-0083-z

**Published:** 2016-01-05

**Authors:** Tobias Hainz, Sabine Bossert, Daniel Strech

**Affiliations:** 1 Johannes Gutenberg University Mainz, Institute for History, Theory and Ethics of Medicine, Am Pulverturm 13, 55131 Mainz, Germany; 2 Hannover Medical School, Institute for History, Ethics and Philosophy of Medicine, Carl-Neuberg-Str. 1, 30625 Hannover, Germany

**Keywords:** Collective agency, Public involvement, Representation

## Abstract

**Background:**

Public involvement activities are promoted as measures for ensuring good governance in challenging fields, such as biomedical research and innovation. Proponents of public involvement activities include individual researchers as well as non-governmental and governmental organizations. However, the concept of ‘public’ in public involvement deserves more attention by researchers because it is not purely theoretical: it has important practical functions in the guidance, evaluation and translation of public involvement activities.

**Discussion:**

This article focuses on collective agency as one property a public as a small group of participants in a public involvement activity could exhibit. It introduces a prominent theoretical approach to collective agents as one specific kind of social entities and demonstrates how this approach can be applied to current practice in public involvement activities. A brief discussion of different types of representation as they are used in the existing literature on this topic is also included because representation and collective agency can be closely related to each other. Suggestions and ideas that are derived from this reasoning include the proposal to use a ‘validity check’ for the generation of collective agents as a regular element of certain types of public involvement activities, the consequences of combining collective agency and representativeness as a further property a public could exhibit, and standards for reporting the content of public involvement activities in scientific publications.

**Summary:**

This article discusses the importance of the concept of ‘public’ in public involvement activities, with a focus on biomedical research and innovation. It introduces various practically relevant ideas that are based on a theoretical analysis of collective agency as an important property a public can possess.

**Electronic supplementary material:**

The online version of this article (doi:10.1186/s12910-015-0083-z) contains supplementary material, which is available to authorized users.

## Background

Public involvement activities (PIAs) are promoted and conducted as a cornerstone of good governance in biomedical research and innovation (BMRI). They appear to be perceived as alternatives to traditional models of governance, such as models that rely on the opinions and evaluation of experts rather than lay citizens. While one might be hesitant to speak of a ‘paradigm shift’, this development is clearly visible and gives the impression that PIAs are more than just a new methodological approach in the social sciences (see, for a similar diagnosis, [1]). Others have, however, adopted the language of paradigm shifts and call this development a “participatory turn” ([2]: 219). Three types of evidence for this impression can be given: the first type is research on PIAs being conducted and reported in scientific publications, for example, several reviews and evaluations of PIAs on issues from BMRI, such as [3] and [4], and proposals how and why PIAs in BMRI should be carried out, such as [5] and [6]. The second type are demands of institutions to increase the number and frequency of PIAs in BMRI, for example, a brochure of the European Commission [7] that outlines key elements of responsible research and innovation, recommendations of the German National Academy of Science and Engineering [8] with regard to science communication in biotechnological research, the proposal of the Nuffield Council on Bioethics ([9]: 67) to develop a “public discourse ethics” for the field of “emerging biotechnologies”, and a guideline of the European Commission [10] that calls for PIAs in the context of biobank research. The third and final type are actual PIAs that have been carried out with an explicit agenda in BMRI, for example, the *BC Biobank Deliberation*, held in British Columbia and reported in [5], a report in [11] of a social experiment on public deliberation with a focus on synthetic biology, and a dialogue among citizens on high-tech medicine, initiated by the German Federal Ministry of Education and Research [12].

All of these and similar documents share the property that ‘public’ is among the most important concepts used in them. While these documents might differ with regard to the weight they explicitly put on this concept, most of them depend on it to convey their central message. However, an examination of these and similar documents shows that there are different ways to employ this concept. But, depending on its particular understanding of ‘public’, the theoretical content of such a document as well as its practical implications change significantly. Moreover, a document could even be inconsistent if it uses different understandings without good reason, or confuses them. Hence, an elucidation of different meanings of the term ‘public’ in the justification, planning, organization and evaluation of PIAs in BMRI is desirable.

Three aspects of the concept of ‘public’ can be distinguished: first, the nature of the target population as the ‘public at large’ has to be clarified. Braun and Schultz [13], for example, present and discuss four major constructions of the public at large: the general, pure, affected, and partisan public. Wickson et al. [14] discuss the public to involve as laity, consumers, or stakeholders. In this paper, the underlying idea of the public at large and the properties of its elements will not be discussed in detail. Whatever construction the target population of a PIA might be, usually not all relevant members of this ‘public at large’ can be involved. Organizers of PIAs therefore have to select a number of participants, which at least to some degree are perceived as representatives of the target population. Representativeness as a property of a group of participants is important to allow organizers and decision-makers to generalize the outcomes of the PIA. The concept of ‘public’, secondly, depends on the applied notion of representation, which at the same time comes along with different measures of sampling. Representation has always been a vital issue for democratic theory (see [15] for an overview). Here, we focus on the rather practice-oriented discussion on representation in PIAs. One significant body of literature is the literature on deliberative and discursive forms of public involvement. During the last decades, these forms have been gaining importance in the field of BMRI [16]. On the basis of this body of literature, one can distinguish between at least five different approaches to representation and corresponding methods of sampling: self-selection, elected/delegated, quantitative, discursive and qualitative representation [17–19]. These approaches are to be considered as mere ideals which help to define and analyze the forms of representation applied in a respective PIA. In a real-world PIA, they hardly appear in their pure form; instead, different forms of representation are usually combined.

The first and second aspect of the concept of ‘public’ both focus on the characteristics of individuals: while the first aspect focuses on properties possessed by elements of the ‘public at large’, the second aspect focuses on the representativeness of PIA participants. There is, however, a third aspect that deals with the properties of the group of participants as a whole: a group can be conceived of as a ‘loose cluster’ of individuals who do not stand in any specific relation to each other, so that the group as a whole does not exhibit any noteworthy properties. This would probably be the case for most groups that are assembled in more or less spontaneous, non-experimental settings, where it is not intended to endow this group with any group-level property. Yet one can also imagine mechanisms that endow a group with a property like collective agency, which is not a trivial one.

Although we will provide a concept of ‘collective agency’ in a later section of this paper that is based on recent literature in social ontology, an explanation of our understanding of this concept may be helpful in order to guide the reader to this section: to our mind, one can approach the concept of ‘collective agency’ by analyzing each of its components separately before combining them. First, a collective is to be understood as a social entity that consists of an unspecified number of individuals who share some properties that allow for their identification as a collective (instead of a ‘loose cluster’ of individuals). Participants in a public demonstration, for example, could be identified as a collective because they apparently share several values or even a worldview. Yet some people waiting for the bus can hardly be thought of as a collective since they only share the property of waiting for the bus. If they indeed shared a worldview, this would be an entirely accidental state of affairs. A non-normative property shared by individuals who form a collective could be conceptual interpretations of certain states of affairs. Examples of states of affairs that allow for conceptual interpretations because they typically involve a lack of consensus regarding definitions of relevant concepts are the ascription of personhood to certain entities (like embryos or non-human animals) or – with regard to the topic of this article – the nature of collective agents. Furthermore, sharing values, conceptual interpretations, or a worldview is also a much more stable property than waiting for the bus, for an individual can – literally in a matter of seconds – lose this property as soon as he or she gets on the bus. Collectives therefore exhibit a certain degree of persistence regarding their own identity in the case the identity of their constituents is changed (see also [20]). Second, an agent is to be thought of as an entity, typically an individual, that can hold attitudes like beliefs, judgments, or values, and is able to act in accordance with them. Agency, therefore, is this particular property of possibly being the subject of such motivational attitudes and being able to act upon them that distinguishes an agent from a non-agent. In other words, and with reference to [21], agency requires an individual to be clearly distinguishable from its environment (and, therefore, identifiable), able to actively shape its environment instead of merely exchanging information, and able to evaluate its own behavior with regard to normative frameworks (desires, beliefs, attitudes), generated by itself. Consequently, a collective agent is a collective as explained above that can also be the subject of attitudes and can perform actions as a collective. While ‘collective *agent*’ refers to the subject of these attitudes and actions, ‘collective *agency*’ refers to the property an entity of this kind possesses. Possessing agency, however, does not imply that the collective actually performs any particular action or holds a specific attitude at any instance. It does only imply that it would be possible for it to do so. It should be noted that the very existence of collective agents has been the subject of skeptical as well as affirmative discussions (see, for example, [22, 23]). While we are aware of these difficulties, we postulate the possibility of the existence of collective agents as a working assumption.

All three aspects of the concept of ‘public’ are crucial for the design, execution, normative significance, and evaluation of PIAs. While several papers discuss the different possible target populations and notions of representation, the question of collective agency and its practical consequences and normative implications for public involvement in the field of BMRI has, to our knowledge, not received much attention. Although certain issues in the PIA context can be related to questions of collective agency – like questions regarding the adequacy and design of PIAs [24, 25] as well as conceptually related issues of collective autonomy and responsibility [26] – we believe that collective agency deserves a self-standing analysis with a proper conceptual apparatus. Therefore, after showing why a careful usage of the concept of ‘public’ is in order, an analysis of its different functions, and a brief discussion of different kinds of representation with the purpose to orient our paper in the broader context of public involvement, we will further attend to the third aspect of the concept of ‘public’ in public involvement: collective agency as a significant property of the group as a whole. Our analysis will start with a theoretical reconstruction of collective agency from the perspective of one of the most prominent accounts of social entities, the account developed by John R. Searle. It will then result in several proposals for PIA practice that may inspire further inquiries with a stronger methodological orientation.

Although the arguments of this paper can be easily adapted to other fields of public involvement, the analysis focuses on PIAs in BMRI because the authors possess an extensive overview of the PIA literature in the field of BMRI. The authors are members of a research group that has conducted a systematic review of PIAs in BMRI [16]. Since PIAs are apparently prevalent in BMRI and not just a niche phenomenon, this limitation should nonetheless be justified.

As a conceptual preliminary, it should be mentioned that beside ‘public involvement’, other expressions are frequently used in a similar fashion, such as ‘public engagement’, ‘public deliberation’, or ‘public participation’ (see, for example, [27, 28]). However, ‘public’ deserves a separate analysis, as do the expressions mentioned above. Our choice of ‘public *involvement*’ as an umbrella term for diverse activities is mainly motivated by its neutrality: while an informational event will be different from a consultative or deliberative event, both events share the property that they (in general) *involve* a group of people that is referred to as ‘a public’. A preference for another umbrella term should not be a reason for disagreement on the central issues of this article. It should also be noted that although the examples of PIAs we use for illustrative purposes usually count as single events or at least as events that lasted for a clearly defined period of time, the relevance of our theoretical discussion and its implications is probably not limited to these kinds of PIAs. Whenever it is imaginable or even purported that a collective agent exists in a PIA context, the reasoning presented in this article should be applicable, even though it could become more difficult to apply it because of the increasing complexity of PIAs with an indefinite duration.

## Discussion

### Why is the concept of ‘public’ important?

PIAs are often designed to either involve the public in governance activities and to thereby introduce an element of ideal-typical modern democracies like deliberation [29, 30] to decision making or to contribute insights and knowledge of the public to decision making [31]. In some cases, PIAs just aim at informing the public about the issue at hand. Depending on the respective objectives of a PIA, the target population has to be identified. Accordingly, the nature of the PIA and its impact vary. Yet not only the objectives of a PIA and the definition of the target population can have practical and normative implications. The respective notion of representation and the perception of the group of participants as a collective agent rather than a ‘loose cluster’ of individuals, for example, can be decisive for the recruitment of participants and the possible impacts of the PIA. Ideally, all of these parts a PIA consists of are derived from its aim, so that an acceptable level of what one could call ‘internal consistency’ is achieved. However, as we will argue and demonstrate below, this internal consistency can also be reduced when one uses the concept of ‘public’ in certain ways that imply the existence of a collective agent while, in fact, the organizers of a PIA have not undertaken any visible efforts to create a collective agent during the PIA process. This kind of reduction of internal consistency can render a PIA much less valuable than it could have been if all of its parts had been adjusted to each other, with the PIA’s aim being the part from which all others are derived. Internal consistency, therefore, is one property of a PIA organizers should try to promote. As we will argue below, the concept of ‘public’ used in a PIA can increase or decrease the internal consistency of a PIA, depending on its usage in each of the PIA’s different parts, such as its aims, its recruitment process, and the methods used in it. While our analysis focuses on collective agency as a specific property a public can exhibit, these remarks on internal consistency do not exclusively apply to this focus but can be generalized.

The question whether the *aims* of a PIA have been achieved cannot be answered without considering the concept of ‘public’ used by its organizers. For example, the statement “Our PIA discovered several values that are shared by a wide range of people with diverse preferences within our society” is only true if the ‘public’ in the respective PIA refers to something that can be regarded as representative of the ‘wide range of people within our society’. Or, to give a further example, it is implausible to claim that a PIA has “given a voice” to a group of participants unless specific efforts have been undertaken that are sufficient for creating a collective agent that actually has a voice of its own.

The PIA’s *recruitment process* also depends on the concept of ‘public’ used. One can, for example, choose whether one recruits for hard criteria that are easily operationalized (such as age, sex, salary, and so on) or whether one includes softer criteria in the recruitment process (such as lifestyle, internalized values, political preferences, and so on). The inclusion of softer criteria might enable organizers of a PIA to create a collective agent more easily inasmuch as the people forming the public could be more willing and able to ‘speak with one voice’ if they share certain qualitative characteristics. For, a group of people who are only recruited because of hard criteria like age or salary may still be very heterogeneous with regard to the lifestyle or internalized values of its members. There could be a tendency to disagree with each other and, consequently, unwillingness to form a collective agent together. On the other hand, sharing qualitative characteristics could motivate people not only to be a part of a mere group but also to be a part of a collective agent and, figuratively speaking, to amplify this agent’s voice. A further, normatively relevant question regarding the importance of the concept of ‘public’ for a PIA’s recruitment process concerns the selection of participants with regard to their affectedness [32]. It remains an open question and might even depend on the purpose of a specific PIA whether only people are recruited as participants who will be directly affected by the phenomenon on the agenda or whether unaffected people who can nevertheless hold an opinion towards this phenomenon should be recruited as well.

Third, the *method* of a PIA depends on its concept of ‘public’. Imagining a panel discussion among experts who can be questioned by lay people attending this event, it would appear strange if the organizers of this type of PIA employed a concept of ‘public’ that referred to more than an arbitrary collection of individuals. Events of this kind are usually directed towards individual persons in order to spread some pieces of information, but they are usually not directed towards a collective agent who is only expected to receive these pieces of information without being able to use it, because the agent ceases to exist at the end of the event.

Hence, the three aspects of the concept of ‘public’ are highly relevant for the organizers of PIAs. Other professionals involved in PIAs, such as researchers who evaluate them or decision makers who refer to their findings, should also recognize its importance. In general, the concept of ‘public’ can be said to have three functions in the context of public involvement, according to the different groups of people to whom it is relevant (see Table 1).

**Table 1:** Functions of the concept of ‘public’

Function:	Directed towards:	Related to:
Guidance	Organizers	PIA’s aims
		PIA’s initial design
		PIA’s recruitment process
Evaluation	Evaluators	PIA’s feasibility
	PIA scientists	PIA’s validity
		PIA’s success
Translation	Decision makers	Translation of PIA’s results

**Table 2:** Conditions for collective agency with regard to publics

Status function	The public was declared to be a collective agent.
Collective recognition	The public is collectively recognized as a collective agent.
Collective rationality	The public’s structure allows for the formation of consistent collective beliefs and desires.

The concept of ‘public’ has a guidance function inasmuch as organizers of a PIA are well-advised to create as much consistency as possible between the concept they use on the one hand, and the PIA’s aims, design, and recruitment process on the other hand. It deserves the same level of attention as, for example, the choice of a particular sampling method. The type of public created in a PIA should not be regarded as a merely contingent and overall negligible result of a combination of a recruitment process and the PIA’s design. It should rather be regarded as a necessary result of the recruitment process and the PIA’s design being a *reasonable reaction* to the concept of ‘public’ initially used by the organizers. Therefore, the concept of ‘public’ has a direct influence on the further elements of a PIA, such as recruitment method and design, so that it guides the organizers towards certain reasonable decisions and helps them to avoid unreasonable ones.

It also has an evaluation function because it helps researchers who externally evaluate a PIA to determine its feasibility, validity, and success. A PIA whose design is adapted to the public purportedly created in it can be regarded as more well-rounded than a PIA that exhibits a rather low level of consistency between these and maybe further elements. If the organizers of a PIA claim that they have created a public with a complex property like collective agency but describe methods that are insufficient for creating this type of public, this PIA should be evaluated negatively with regard to its feasibility. Finally, the success of a PIA also depends on the concept of ‘public’ used by the organizers, so that evaluators can investigate whether the aims purportedly achieved by a PIA *can* actually be achieved by using the respective concept.

Decision makers who wish to take the result of PIAs into account can benefit from the translation function the concept of ‘public’ has. A translation of a PIA can be any attempt to shape the ‘real world’, that is, the world outside of the PIA setting with reference to the PIA’s results, typically by using these results as a justification. The translational implications of a PIA can vary, and it partly depends on the concept of ‘public’ used in it. Some PIAs may involve a public that is representative of a larger population, so that these PIAs could provide justifications for decisions affecting this population, whereas other PIAs that do not involve a public with this property should be handled with care by decision makers. Translation of a PIA can have many different facets, and it goes beyond the scope of this analysis to describe them in detail. Yet one can imagine examples that illustrate how translation can take place. One example is a report of a regionally conducted PIA that is used by a local advocacy group in a campaign for their agenda. If this advocacy group claims, with reference to this PIA, that the regional public actually supports their agenda, this claim is only true if the organizers of this PIA ensured that there is some kind of representativeness relation between their sample and the regional public. A second example could be a government that attempts to translate the results of a large citizen conference into a legally binding guideline. This translational effort appears to be much more legitimate if the public involved in this citizen conference had some kind of mandate to provide translatable recommendations, for example, by being able to ‘speak’ for a larger population. A third example that is different from the previous ones because it does not concentrate on regulatory efforts could be the educational impact of a PIA, for example, because of the information delivered to the participants or because of the participants’ self-experience of preference changes within debates.

We will now give three examples that, in our experience, are typical for the lack of explanation and justification regarding the concept of ‘public’ and the properties asserted of the particular public involved. We chose these examples for didactic reasons only and are aware of several similar cases.

The report “Generation Scotland: consulting publics and specialists at an early stage in a genetic database’s development” [33], a report on a consultation activity regarding the implementation of a genetic database in 2003 and 2004, states that: “similar to the publics, the specialists also thought GS to be ‘a good idea’.” ([33]: 142) This wording implicitly claims that one can consult specialists (individual entities) in the same way as one can consult publics (social entities) with regard to a certain issue, such as a DNA database. If a PIA aims to involve a public with agential properties, such as being able to think or being consulted (similar to the involvement of specialists), there should be a justification of this surely non-trivial ascription of properties.

A second example can be found in a report on a PIA conducted in 2008 that used focus groups to discuss synthetic biology and the influence of media information uptake as well as deliberation on the formation of opinions [11]. The authors frequently use the expression “groups of the public” ([11]: 184 f.) and implicitly ascribe agential properties to these groups, such as focusing on specific kinds of information ([11]: 175), having interests ([11]: 177), or being supportive of synthetic biology ([11]: 183). Yet it would be more satisfactory for the reader if the authors not only asserted that the public has these properties and that a part of it, the groups, have them, as well, but also provided a brief justification.

It seems that either authors of PIA reports frequently use the term ‘public’ on purpose without bothering with the underlying assumptions and their consequences, or use it without considering alternative expressions. Both explanations, however, call for a correction of this current state of affairs, because of the strength of the normative rationales for PIAs.

Finally, Menon and Stafinski [34] report the results of a citizen jury on priority-setting for health technology assessment. They repeatedly ascribe agential properties to the public without specifying why they have reasons to believe that the public they refer to actually has these properties. This is especially noticeable when they suggest that the public can regard something as a value in the same way as individual persons (in this case, decision makers) can regard something as a value ([34]: 291). This suggestion does not only imply some specific value distribution across a population but that there is a further entity, the public, that also has attitudes towards values, which should probably be taken into account. It also raises the question why the public and decision makers form a dichotomy, because one could believe that decision makers are part of the public and not something independent from it.

### Different kinds of representation

As noted above, there is a broad and ongoing discussion within PIA literature (see, for instance, [5, 13, 14, 25] on the properties of the intended target population as well as on different notions of how participants represent the public at large. Here, we will give a short overview on five ideal-typical notions of representation, which can be distinguished according to the practice-oriented literature within the well-established field of deliberative and discursive forms of public involvement. Since this paper focuses on collective agency, the purpose of this overview on representation is not to provide any particular insights on this topic itself but to be a preparatory section for an analysis of the relationship between collective agency and representation, which will be conducted in a later section.

Individuals can be elected or delegated by social entities or by third parties to represent particular subgroups of the public at large. This type of representation is called *elected representation* [18, 19]. Although any type of representation is hardly ever applied in its pure form in a PIA, an example of such a cluster of representatives can be found in [35], where the results of a multi-staged study on the views of African immigrant community leaders on genomics and biobanking are reported. The first two stages of this study consisted of focus groups and semi-structured interviews, respectively ([35]: 197). These stages clearly involved individuals with some (socio-political) authorization to represent their community, as the following statement regarding limitations of the study suggests: “Participants were purposely selected because of their stature and level of influence in the black African immigrant community. Their views cannot be taken to represent all their constituents, but rather reflect their roles as community guardians.” ([35]: 202)

Irrespective of this example, in PIAs on BMRI, elected representation is less common than in other fields of governance. Somewhat more common is the second type of representation: *self-selection*. In this scenario, active individuals are perceived to represent inactive ones [18, 19]. This notion of representation, for example, is applied when participants are invited by means of newspaper advertisements or via mailing lists or when they are recruited by means of a convenience sample. One example of a PIA in BMRI that involved a self-selected group of participants is reported in [36]: this paper reports a set of practical exercises for laypeople that were designed for explaining the value of experiments on *Drosophila melanogaster*. The PIA was conducted during a science festival, and every visitor to this festival was, in principle, given the opportunity to take part in the PIA. It had both an informational and a consultative element, because the participants could learn something about the importance of research on flies and because they could provide the organizers with feedback through a questionnaire. Moreover, almost all PIA-organizers invite participants to take part voluntarily; in these cases, the recruitment process inevitably includes self-selective elements.

The third type aims to create a statistically correct image of the public at large, usually by randomly selecting participants. To allow for proportional representation of predefined (socio-politico-demographic) subgroups, quotas of the respective characteristics (such as sex, income, education, religious belief, or political attitudes) are occasionally applied [18, 19]. As this type refers to the frequency of relevant characteristics in the sample and the population, respectively, we call it *quantitative representation*. It is also referred to as ‘statistical representation’ (see, for example, ([18, 37]: 241), ([38]: 112)) or ‘descriptive representation’ (especially [19]). In other contexts, both expressions are used for different kinds of representation. Therefore, we prefer the term ‘quantitative representation’. A typical PIA in BMRI that involved a quantitatively representative collective is reported in Menon and Stafinski [34]. Quantitative representation is one of the most common types, but it entails a major problem: even by stratified random selection, it is hardly possible to create a statistically correct image of the (often large) target population in the (usually much smaller) group of participants.

The fourth type, *discursive representation*, has been developed to solve this ‘scale-problem’ of quantitative representation. In contrast to the three aforementioned types, *discursive representation*, does not aim for representation of individuals or groups but rather of the usually smaller number of diverse views and experiences [39] or of relevant discourses on the issue at hand [17, 40]. These discourses can more easily be represented by a small number of participants. But with this type, some new – as yet not entirely resolved – problems emerge: to achieve discursive representation, organizers do not only need to distinguish all relevant views and discourses in advance, but also to identify and recruit individuals representing the views and discourses. Hence, although there are examples of PIAs in BMRI which aim for discursive representation [11, 41], this form of representation is not easily achieved.

The fifth type of representation offers a pragmatic solution for the challenges of quantitative and discursive representation by combining the objectives of both versions. This type aims for a qualitative diversity of participants instead of a quantitative proportionality [42] and can therefore be referred to as *qualitative representation*. It is based on the assumption that participants with diverse socio-demographic characteristics entail different backgrounds and experiences and hence represent different views, experiences, and discourses. Qualitative representation is usually achieved by some kind of purposive stratification of random samples. It requires that the (sub-)public includes the ‘full spectrum’ of the relevant characteristics from the population (e.g. all types of religious belief within a certain population). A qualitatively representative sample is, therefore, what Brown calls a “societal cross-section” ([43]: 220); yet we prefer the terminology proposed here in order to make clear that qualitative representation is not entirely different from but just one specific kind of representation. For the generation of a qualitatively representative (sub-)public, it is unnecessary that the quantitative distribution (frequency) of relevant characteristics amongst its members matches their quantitative distribution in the population. An organizer of a public participation activity who aims to include a public of 20 people that qualitatively represents the diverse religious beliefs in the relevant region would realize this goal by including at least one individual for each existing religious belief. The term ‘diversity’ is often used in the PIA literature to describe what we (for didactic reasons) call ‘qualitative representation’. PIAs that involved a qualitatively representative sample are reported in [27] and [44].

### Collective agency in theory…

Collective agents, their properties, and the conditions for their existence are a prominent topic in social ontology or action theory, and also in the more practice-oriented field of political philosophy. Social ontologists analyze the ontological properties of collectives and their relation to entities of other kinds (see, for example, [45, 46]); action theorists examine the relationship between agency and collectives (see, for example, [47, 48]); and political philosophers analyze the relationship between collective agency and democracy (see, for example, [49]), and also between collective agency and responsibility (see, for example, [50, 51]). Our analysis of the concept of ‘public’ as referring to a collective agent in the following paragraphs will be based on the philosophical analysis of collectives in general, but will be limited to the most basic aspects.

A prominent approach to the analysis of social entities can be found in [52], where John R. Searle attempts to reconcile a naturalistic worldview with the purported existence of social entities. According to Searle, social entities are created by a linguistic operation, namely the declaration that a respective status function exists ([52]: 93). Declarations have a general form, given by Searle as “X counts as Y in C”; yet this form can be modified and specified in different contexts, especially with regard to the ascription of normative or “deontic” (as Searle calls them) powers to certain individuals ([52]: 99-102). In other words, given a proper declaration, a thing X counts as some Y in a specific context C, so that Y is X’s status function in this context C. A status function is a function that is imposed on an object or a group of objects. An example is the function of being money that is imposed on a piece of paper, but only in the context of trading, or the function of being the president of the United States that is imposed on Barack Obama, but only in the context of his actual presidency. One can therefore imagine contexts where a piece of paper does not count as money, for example, in a context where no trading is possible. There could also be a context in which it is impossible to assign the status function of being the president of the United States to Barack Obama, namely, a context where another person has already been assigned this status function. As Searle ([52]: 7) also notes: “The performance of the function requires that there be a collectively recognized status that the person or object has, and it is only in virtue of that status that the person or object can perform the function in question.” This implies that in some cases, a declaration is insufficient for an X to count as a Y in a context C, namely, when collective recognition of the validity of this declaration is absent. If, for example, one tries to use some piece of paper as money, even in a context of trading, there could be a lack of collective recognition that *this* specific piece of paper counts as money, rendering it virtually useless in this context.

Now, one may ask *who* can create a social entity by means of a declaration, and *whose* collective recognition of the validity of this declaration is necessary. Since Searle seems not to be explicit on this issue, one has to rely on an implicit statement he makes when discussing the transformation of a simple, physical wall into a socially significant boundary: “the people involved” ([52]: 94) need to collectively recognize the counting of the wall as a boundary. This very vague formulation can be rendered more precisely by stating that only a group of people who will be perceivably affected by a social entity – instead of an arbitrarily chosen group – can collectively recognize its existence. Consequently, one should also assume that only people who are involved in an attempt to create a social entity can make an effective declaration. The identity of these people, of course, differs with regard to different social entities, but in the context of collective agents, it appears reasonable to assume that only people who are thought to be parts of a collective agent or at least someone who can legitimately speak on their behalf can jointly declare it to exist. Since, for trivial reasons, those people will also be affected by the collective agent, their collective recognition should also be necessary.

Another important component of Searle’s approach to social entities is collective intentionality inasmuch as not *individual* but *collective* recognition (which is a kind of collective intentionality) is necessary for an object to have a status function: “In the creation of human institutional ontology, collective intentionality and the assignment of function go hand in hand, because the crucial functions in question require collective intentionality. From a theoretical point of view it is possible for an individual to construct a ‘private’ institution and ‘private’ institutional facts for his or her own usage. For example, an individual might invent a game that only he plays. But the cases important for our investigation, for making the social world, cases such as money and government, require collective intentionality.” ([52]: 59 f.) This statement reflects that for a *social* instead of a *private* entity to exist after a declaration has been made, people who would be affected by this entity need to collectively recognize its existence as this very social entity. This means that they do not only have to recognize (and possibly ignore) that a declaration has been made but they also have to acknowledge the effectiveness and legitimacy of this declaration. An indicator for this acknowledgment as a form of acceptance could be adherence to the rules explicitly or implicitly formulated in the context of the declaration: referring to Searle’s example, as soon as the person who creates a game wishes to play it together with others, they have to collectively recognize the social components of this game, such as its rules and its winning conditions, and act in accordance with them. If they just move some tiles across a board in an arbitrary fashion but ignore the inventor’s constant complaints, they may have collectively recognized the existence of a declaration, but not its effectiveness in creating a game as a social entity. Searle’s account of collective intentionality is rather technical and involves notions like ‘belief’, ‘causation’, and ‘constitution’ ([52]: 50–55), but it can be boiled down to the following statement: collective intentionality is irreducible to individual intentionality, that is, it cannot be analyzed solely in terms of individual intentionality, but it nonetheless exists only in the heads of individuals, not in some mysterious location outside their heads. Therefore, according to Searle, collective intentionality is a genuine feature of the natural world. Furthermore, one can collectively intend to perform a collective action, but one cannot individually intend to perform a collective action.

In short, it is possible to impose a status function on an object by means of a declaration made by people who are involved in the creation of this social entity. Yet this does not imply that this object, in virtue of its status function, is already recognized as a social entity. It is additionally required that this object is collectively recognized by those who would be perceivably affected by it as having this status function. Therefore, a declaration and collective recognition by, to recapitulate Searle’s formulation, the people involved are both necessary and jointly sufficient conditions for the creation of social entities.

This brief treatment leaves a couple of open questions that cannot be answered here, for it would require a more detailed discussion of social ontology that is not needed for the main line of reasoning in this article. One could ask for the specific role of an individual within a collective when it comes to the generation of collective actions that are somehow dependent on but not reducible to individual actions. A possible response – but probably not the only reasonable one – could be that individual actions are necessary and jointly sufficient for the generation of collective actions but that as soon as it is impossible to ascribe causal responsibility for the results of these actions to one individual, one should acknowledge the existence of a collective action. A further open question concerns causal relations between a collective and the individuals being parts of it: is it possible for a collective to have a causal influence on the attitudes of the individuals forming it without ceasing to exist as this very collective? We believe that this is possible as long as, first, the influence on an individual’s attitudes cannot be causally ascribed to another individual but only to the collective as a whole and, second, there is a sufficient degree of consistency between the collective’s actions before and after the causal influence on the individual’s attitudes has been exerted. These hints to open questions should show that there are many theoretically interesting issues to be dealt with, especially because of their relevance for the context of public involvement, which is still a practical one.

Searle’s account of social entities is by no means the only account. However, we prefer Searle’s account to others because it is probably the most elaborate philosophical analysis of social entities and, as a consequence, can be applied to more specific questions without much further work. Furthermore, as we will show later, its theoretical elements map well to practical elements of PIAs.

It should be noted that being a collective does not entail collective *agency*, which requires more than just being a collective in the Searlian sense. As List and Pettit ([53]: 36 f.) claim, a collective has to exhibit a certain degree of rationality in order to be regarded as an agent. (In fact, List and Pettit prefer ‘group’ over ‘collective’, but this is a mere terminological difference, and we wanted to avoid unnecessary confusion by introducing more terminology.) Without going into detail, this degree of rationality can be ensured by designing the collective in a way that allows for the formation of consistent collective beliefs and the satisfaction of collective desires, both of which should be based on the beliefs and desires of its members. If these conditions are not satisfied, this counts as evidence against the collective’s being rational and, probably, its status as an agent.

It should now be clear that there is a set of conditions that need to be satisfied for a collective agent to exist if one subscribes to Searle’s account of social entities in general and to the additional rationality condition. As one can see, collective agency appears to be normatively neutral, but it can quickly become a normatively important property of a collective in a context like PIAs and their relation to issues of political legitimacy, but also because it is conceptually related to a normatively relevant property like collective autonomy. If one believes that a collective agent is also collectively autonomous, one apparently subscribes to at least two normatively relevant beliefs ([26]: 292–295): first, this autonomous collective agent is not only able to hold attitudes that are partly independent of the attitudes of its members, but it is also justified in overriding dissenting opinions of its members. Second, one can ascribe rights as well as duties to this autonomous collective agent, so that these rights and duties do not ‘trickle down’ to its members. This implies that, for example, demands raised by this agent may deserve the same consideration as demands raised by individual people, but also that this agent can be held accountable for actions that are performed in its name. While it remains an open question that we cannot discuss here any further whether it is a conceptual truth that collective agency entails collective autonomy, one should take these considerations seriously because they can quickly become relevant in the PIA context.

For those readers who are interested in the conceptual and methodological difficulties one can encounter when creating a collective agent, we included an additional file that introduces the so-called ‘discursive dilemma’. This dilemma lies at the center of a growing research field dealing with *judgment aggregation*, that is, the aggregation of individual to collective judgments. By introducing this dilemma, we would like to illustrate that even in a context like majority voting – which is usually less complicated than most PIA contexts – there are formal challenges that need to be addressed before one can confidently claim that one has succeeded in creating a collective agent. Consequently, if these challenges arise in a context of comparably low complexity, one can expect to encounter those or even more difficult challenges in typical PIA settings (Additional file 1).

### …and in practice

The following table recapitulates the account of collective agency developed in the previous section and provides a rough summary of the three crucial conditions for an entity to be a collective agent. While the assignment of a status function and the collective recognition of the entity’s existence are necessary for interpreting it as a social entity at all, it also needs to exhibit collective rationality with regard to the formation of its beliefs and desires or, in short, attitudes. If it does not exhibit a sufficient degree of collective rationality, it might be called a ‘collective output machine’, but it does not qualify as an agent (Table 2).

One example of a collective agent featured in a PIA is the public that was involved in a deliberation event reported in [54]. This PIA employed methods of deliberative democracy and resulted in several statements that could assist representatives of biobanks in developing policy guidelines. One important feature of their PIA is stressed by ([54]: 1607): “Analyses therefore need to differentiate between individual opinions expressed in discussion, themes emerging from analyses of the entire discussion, and collective statements ratified by the group. We term these collective statements ‘deliberative outputs’. In contrast to post hoc analyses of deliberation transcripts, deliberative outputs have additional political legitimacy because they represent collective positions arrived at through democratic deliberation, that are subsequently ratified as such.” In other words, the participants of the PIA did not just express their individual opinions at the end of the event – they also formed a collective with its own opinion in the form of a deliberative output. This is a descriptive statement, but it is accompanied by the normative statement that the deliberative output has special political legitimacy: for, not only is the output especially praiseworthy from a procedural perspective that values deliberation as a democratic ideal (for overviews on the relationship of deliberation and democracy, including its challenges, see [55, 56]), it is also ascribed as an opinion to a collective entity whose political status goes beyond the political status of mere individuals.

Applying Searle’s account of social entities to this PIA and the claims of its organizers, one can formulate the following conditions which have to be met for these claims to be true. First, there must have been a declaration, ideally jointly made by the organizers and the participants, that the individuals involved in the PIA actually form a collective agent – they must have been assigned the respective status function. It should be noted that a declaration does not always have to be an utterance of a specific form. What is important is that some operation takes place that assigns a specific status function – being a collective agent – to the group of people participating in a PIA. This can be a verbal agreement that one intends to act as a collective that overrides individual opinions. Yet this can also be a set of rules for discussions among the participants, so that the content of these rules implies the existence of a collective agent without claiming that it exists explicitly. Second, it has to be collectively recognized that these people have this status function – not least by the individuals themselves. Third, the “collective positions” or “deliberative outputs” should satisfy certain rules for consistency, so that encountering a discursive dilemma or other constellations that indicate a lack of consistency can be avoided.

The first and second condition can certainly be met, and one can believe that the organizers of the PIA collectively recognize the public they involved as a collective agent. This belief is especially justified because the organizers describe that the collective positions formulated in a process of deliberation were subsequently “ratified”, which can be interpreted as a PIA-internal control mechanism. By using this mechanism, the organizers check whether the participants regard themselves as parts of a genuine collective agent with attitudes of its own and not just as individuals who do not form a collective agent. However, one can ask whether this collective recognition on the part of the organizers is sufficient for them actually to be a collective agent. This question targets not only the PIA in question but Searle’s whole account of social entities, because it prevents a clear response to the question of how strong the collective recognition of a declaration has to be. Suppose that there is a group of biobank opponents who deny that the people who were involved in the PIA’s public had the status of a collective agent. While the opponents could still acknowledge that the PIA’s output is the result of a deliberative process, they could deny that this result can be interpreted as the opinion of a collective agent. In this case, the declaration of the organizers of the PIA and the public’s collective self-recognition is confronted with a collective denial of the declaration’s effectiveness. This conflict appears to be difficult to solve as long as no specific and feasible proposal regarding the necessary strength of collective recognition exists.

There are two reasons why we believe that the collective recognition by the organizers alone is likely to be insufficient to create a genuine collective agent during a PIA. First, it would imply an odd diffusion of normative power to all kinds of PIA organizers. People, for example, a small sociological research group, would just need to organize a PIA and collectively recognize the participants as a group forming a collective agent in order to satisfy one of three conditions for the creation of such a collective agent. The normative power these people would be endowed with would not reflect their probably low status when it comes to questions of democratic legitimacy. Second, it would also imply that it could be quite easy to actually create a collective agent, which should be thought of as a comparably complex entity. This could lead to a multiplication of collective agents beyond any reasonable degree.

While it may be possible that the third condition has also been met in this PIA, we cannot know this for certain because this would require an in-depth analysis of requirements for the formation of consistent collective judgments in public deliberation settings. It is obvious that a discursive dilemma as described in the additional file is unlikely to occur in a PIA like this because deliberation is different from majority voting. However, it could be the case that a public deliberation setting can produce results that are similarly unsatisfactory as the discursive dilemma. While we cannot discuss it in more detail here, we would like to mention that under certain plausible conditions it is impossible to transform judgments into other judgments that are not identical to the previous ones – in other words: if these conditions are satisfied, no opinion change occurs (for an extensive discussion of this phenomenon, including a formal proof, see [57]). Yet since the transformation of individual judgments in order to arrive at a consensus is the very aim of many deliberative PIAs, this phenomenon could pose a threat to the purpose of such PIAs. However, assuming that the organizers were successful in meeting the third condition, one can regard their PIA as featuring a genuine collective agent.

As this example shows, theoretical reasoning about the properties of publics as collective agents can be transferred to the practice of PIAs in BMRI. We will now provide some further, more general ideas and suggestions regarding collective agency and its relationship to other aspects of public involvement. These ideas and suggestions should not be regarded as conclusions that are set in stone but as reflections that are generated from the reasoning previously presented in this article and are open for refinements.

#### Validity checks

How can PIA organizers argue with justification that the public involved functions as a collective agent?

In practice, a kind of validity check might help analyze whether the people who are involved in a PIA indeed think and act as a collective agent. To assess this ‘internal’ validity, the PIA participants should discuss whether they regard and ‘declare’ themselves as a collective agent. The assessment should be repeated after the PIA’s outcome is settled. Do the participants understand the PIA’s outcome as a product that they generated collectively? Besides the internal validity check, some outside look at the same issues might be important as an ‘external’ validity check of the conditions for collective agency and the closely related conditions for democratic legitimacy. For, at least some prominent approaches to democratic legitimacy incorporate ideals – such as rational deliberation as a means to arrive at a favorable outcome [58] – that are similar to requirements for the creation of collective agents. Some other approaches even neglect outcomes and focus entirely on the procedural virtue of deliberation as the source of democratic legitimacy [29, 59]. This view closely resembles the view that certain structural relations between individual attitudes, as opposed to their actual content, are a vital component of collective agency. However, many open questions regarding feasibility, acceptability, and validity of this sort of check arise, and it is beyond the scope of this paper to address them adequately. Our proposal for a validity check should be read as an attempt to further critical reflection, discussion, and pilot studies on the issue of collective agency in PIAs. In our opinion, further research could hold the result that validity checks should be regular components of any PIA that purports to involve a collective agent. This implies that the idea of collective agency would generate a novel procedural component of certain PIAs. This should, therefore, be considered as a motivation for theoretical reasoning about the concept of ‘public’ because it would have been shown to be directly relevant for PIA practitioners. A further problem arises in this context that can be related to the questions *who* can declare a social entity to exist and *whose* collective recognition of its existence is needed, which were asked in the previous, theoretical section on Searle’s account of social entities: if the organizers and some participants in a PIA declare the respective public to be a collective agent, but some dissenting participants object that they do not regard themselves as parts of this collective agent (for whatever reason) – how should this public be conceptualized?

#### Collective agency *and* representation?

One should note that a public can be a collective agent and still have further properties that specify the kind of public it is. In other words, collective agency and other specifying properties are not mutually exclusive. This is particularly relevant for the case of groups of participants who stand in a specific representativeness relation to the public at large. A special kind of public could share the properties of being in some way representative of a larger population *and* being a collective agent. What does this imply?

Considering elected representation, one could infer that a public that consists of several elected participants and is also a collective agent is not only *able* but genuinely *entitled* to speak on behalf of the electors. Being entitled is the direct result of being constituted by elected participants, and being able is the direct result of being a collective agent. A public that is both a collective agent and a collective of elected participants could, therefore, exhibit a significant degree of normative force. Its decisions or recommendations could be regarded as normatively binding for both the electors as well as those towards these decisions and recommendations are communicated.

Similar reasoning also applies to the combination of collective agency and quantitative representation: a public that is a collective agent and quantitatively representative of a larger population, maybe even a whole society, would be able and entitled to speak on behalf of this population. This implies that one would be justified in inferring any descriptive statements about the attitudes of the larger population from statements uttered by the smaller public. Furthermore, whatever decisions or recommendations the smaller public would communicate should be regarded as normatively binding.

A discursively representative collective agent might not stand in a noteworthy relation to a *larger population itself*, but at least to the *discourses within a larger population*. It would be able to communicate something that could be regarded as a minimal consensus among all of these discourses. Recommendations provided by a public of this kind could be interpreted as necessary conditions any future regulations or decisions by policy makers need to satisfy in order to reflect this minimal consensus.

This possibility of combining collective agency and a form of representation again shows why it is important to consider the relationship between collective agency and collective autonomy: if one believes that this relationship is very tight, one has reasons to believe that a quantitatively representative public that is also endowed with collective agency and, consequently, collective autonomy has a *right* to act as a delegate of a larger population. Yet on the other hand, this collectively autonomous public can also be held accountable for actions performed by the larger population if it has certain duties and if it stands in a proper representativeness relation to this population. Therefore, creating a collective and representative agent is not only conceptually and methodologically intriguing but also a normatively significant endeavor if one takes collective autonomy into account.

Note that being representative and being a collective agent are jointly sufficient for regarding the public’s attitudes as the attitudes of the larger population, but that none of these properties alone allows for this interpretation. The claim that the views of a representative public reflect the views of the larger population (see, for example, [60]: 127) is unsubstantiated as long as no efforts have been put into endowing this public with collective agency. On the other hand, if a public can be regarded as a collective agent, it can be regarded as having attitudes of its own, but as long as it is not representative of a specific larger population, these attitudes should not be interpreted as the views of this population.

Two limitations to this idea of combining collective agency with forms of representation need to be mentioned: first, it seems most plausible to combine collective agency with elected, quantitative, or discursive representation because combining it with one of the other two forms appears to hold less significant consequences. A self-sampled or qualitatively representative public does not stand in any noteworthy relation to a larger population, which is why even if a public of one of these forms could still be a collective agent, it would not be able to speak on behalf of anyone else than itself. A self-sampled public that is also a collective agent could even be criticized for posing as an entity with a voice of its own that need to be taken seriously while, in fact, it can only be legitimately regarded as an entity with one ordinary voice among ordinary others (because it does not relate in any particular way to a larger population). A qualitatively representative collective agent would be remarkable because it would demonstrate that it is possible to unite people with very diverse backgrounds in one collective entity. Nonetheless, even if an entity of this kind existed, its attitudes would still be questionable from the perspective of normative bindingness because these attitudes would not reflect the attitudes of anyone else but the entity itself. It would merely show that diverse backgrounds do not have to be a barrier when one tries to create a collective agent from individuals. Second, since it is practically impossible to achieve the maximum level of quantitative representativeness in the context of typical PIAs, one should be careful to ascribe this ability to a public without reservation. A precise formulation of the relationship between different levels of quantitative representativeness and claims about the normative bindingness of a public’s attitudes might make a valuable research topic.

#### Internal consistency of PIAs

We already mentioned internal consistency as a major requirement for valuable PIAs. A PIA is internally consistent, with respect to the concept of ‘public’, if in each of its parts (that are ideally derived from its aim), the concept of ‘public’ is used identically. It should now be clear that this is a non-trivial requirement since a public may or may not be a collective agent and, consequently, may or may not be thought of as an entity with attitudes of its own. We wish to make the following suggestions that might be of assistance when PIA evaluators as well as decision makers try to assess the internal consistency of PIAs with regard to the concept of ‘public’. It is important to check whether the aim of a PIA can be achieved by the public purportedly involved in this PIA. The wording used in the formulation of the aims can often be used as an indicator of some specific requirements the public has to meet for this aim to be achievable. If, for example, the aim of a PIA is ‘to consult the general public’, this implies that the public involved in the PIA needs to be both quantitatively representative of the general public and a collective agent. To give another example, a PIA that merely wishes to ‘explore opinions within the public’ does not need to involve a collective agent because exploring opinions that are not the opinions of a group as a whole does not require the property of collective agency to be exhibited by this group.

After the requirements the public has to meet have been extracted, the PIA’s internal consistency can now be assessed by analyzing whether the PIA contains mechanisms that ensure that its aims can be achieved. Mechanisms of this kind could be a validity check as described above but also particular sampling methods that are sufficient for achieving, for example, elected or quantitative representation.

This demand for internal consistency shows the need for organizers of PIAs not only to have a sufficient understanding of the possible conceptual issues around involved publics in order to design and conduct their PIAs, but also to report them appropriately. The successful translation of PIA findings into practice depends on the ability of decision makers to identify PIAs that possess the internal consistency to deserve translation. Therefore, reporting the conceptual issues appropriately is not only of interest for research-based evaluations of PIAs but is also a means to assist policy makers in arriving at justified decisions.

### Limitations

One limitation of our analysis is that its examples focus on the specific context of BMRI, and neglects PIAs in other contexts. However, this context-specific limitation is no disadvantage, because the theoretical analysis and its results are independent of the context to which it is applied. Our motivation to focus on PIAs in BMRI was the apparent lack of discussions on collective agency in this field which, in our opinion, needs to be addressed. A second limitation is that we focused on one specific conception of collective agents, which relies on Searle’s account of social entities. An analysis of collective agents from a different perspective might arrive at different theoretical results and equally different practical implications. What we discuss is, therefore, one *possible* and – given the prominence of Searle’s account – *prima facie acceptable* application of a specific ontological theory to PIAs, but we do not suggest that this is the *best* way of interpreting PIAs from an ontological point of view. It should also be noted that while our analysis has practical implications – which we discussed in the previous section – it is not ready for immediate application. There should, however, be enough suggestions with a sufficient degree of both theoretical background and practical relevance that could inspire less conceptually, but more methodologically oriented discussions of collective agency and PIAs in the BMRI context.

## Conclusions

PIAs have grown to be a visible part of BMRI. This article analyzed a purposively selected set of conceptual issues to demonstrate their theoretical and practical relevance. Organizers and evaluators of PIAs as well as decision makers who consider PIA outcomes should put a more explicit focus on collective agency and its relevance for the concept of ‘public’ in public involvement. This could lead to more acceptable PIAs, reports, and evaluations, as well as policy decisions. In order to achieve this aim, we suggest that validity checks could be integrated as a regular element in some forms of PIAs that purport to involve a collective agent. How these validity checks could be designed and evaluated is a subject for further research. The relationship between collective agency and other properties a public could exhibit also deserves to be investigated in detail. While combining collective agency with certain forms of representation appears to be a highly valuable endeavor in certain circumstances, other combinations should be explored, as well. Since successful evaluations of the internal consistency of PIAs strongly depend on the way how they are reported, PIA organizers should be aware of the conceptual issues raised by the claim that a PIA involves a collective agent and report them appropriately. Researchers who evaluate PIAs would benefit from appropriate reports as well as decision makers who attempt to translate them into practice.

## Additional file

Additional file 1: Table S1. The discursive dilemma in biobanking. This additional file provides further information on a theoretical problem in judgment aggregation, known as the ‘discursive dilemma’. First, the discursive dilemma will be introduced in a context-neutral way before its relevance will be demonstrated in an example referring to the context of biobanking [61, 62]. (DOCX 17.4 kb)

## Abbreviations

BMRI: Biomedical research and innovation
PIA: Public involvement activity
