# Supplementary material for: Collective agency and the concept of ‘public’ in public involvement: A practice-oriented analysis
Source: BMC Med Ethics. 2016 Jan 5;17:1. doi: 10.1186/s12910-015-0083-z (PMC4702418; doi:10.1186/s12910-015-0083-z)
Supplement: Additional file 1: Table S1. — The discursive dilemma in biobanking. This additional file provides further information on a theoretical problem in judgment aggregation, known as the ‘discursive dilemma’. First, the discursive dilemma will be introduced in a context-neutral way before its relevance will be demonstrated in an example referring to the context of biobanking [61, 62]. (DOCX 17.4 kb) [file 12910_2015_83_MOESM1_ESM.docx]

The discursive dilemma arises when one attempts to create a collective judgment by majority voting while, however, the voting procedure generates a judgment that is inconsistent relative to the individual judgments. The original discussions of the discursive dilemma can be found in [[58](#_ENREF_58)] and [[61](#_ENREF_61)]. For a fairly accessible review of recent research on judgment aggregation, including the discursive dilemma, see [[62](#_ENREF_62)]. We will use the structure of the example provided there but modify its content, so that it is situated in the field of BMRI.

Imagine a jury consisting of three members, denoted as ‘(1)’, ‘(2)’, and ‘(3)’, who have to vote on three propositions, denoted as *p*, *q* and *p*→*q*. *p* is a placeholder for the statement “Children are unable to consent to storing their tissue samples in biobanks”, *p*→*q* is a placeholder for “If children are unable to consent to storing their tissue samples in biobanks, the storage of children’s tissue samples in biobanks should be prohibited”, so that *q* is a placeholder for “The storage of children’s tissue samples in biobanks should be prohibited”. Although this example is artificial, it is certainly not too far away from real ethical problems one faces when discussing the eligibility of children as donors of tissue samples. As a matter of fact, the members of our jury vote on these propositions as depicted in the following table, generating the following majority judgments.

Table S1 The discursive dilemma in biobanking

|  | ***p*** | ***p→q*** | ***q*** |
| --- | --- | --- | --- |
| **(1)** | True | True | True |
| **(2)** | True | False | False |
| **(3)** | False | True | False |
| **Majority** | True | True | False |

As one can see, each member of the jury holds consistent judgments regarding the eligibility of children as donors of tissue samples, giving a recommendation (*q*) that is justified by the judges’ further beliefs (*p* and *p*→*q*). However, the majority, when interpreted as a collective agent, holds inconsistent judgments because, by the rules of propositional logic, one cannot consistently believe that *p* and *p*→*q* are true while *q* is false.

It should be stressed that the simplicity of this example does not speak against its practical relevance: if one can encounter the discursive dilemma in situations that feature just two simple propositions that are connected with only one logical operator, it is even more likely to encounter it in situations featuring more complex propositions and a wider variety of logical operators. Since PIAs in BMRI typically involve more than two and logically complex propositions, one can assume that collective agents holding inconsistent judgments can be more common in actual PIAs than in artificial settings like the example in table 2. As List ([[62](#_ENREF_62)]) shows, it is possible to avoid encountering the discursive dilemma by relaxing certain conditions that are usually imposed upon majority voting as a common generation mechanism of collective judgments. We will not delve any deeper into this discussion because it would take us too far away from the subject of this article. One should, however, acknowledge that creating a collective agent that is able to hold consistent judgments is no trivial affair at all but can be difficult even in very simple settings that are much less complex than actual PIAs.
